# Impact of perceived distances on international tourism

**DOI:** 10.1371/journal.pone.0225315

**Published:** 2019-12-04

**Authors:** Trivik Verma, Luís Rebelo, Nuno A. M. Araújo

**Affiliations:** 1 Faculty of Technology, Policy and Management, Delft University of Technology, Delft, the Netherlands; 2 Institute for Terrestrial Ecosystems, ETH Zürich, Universitätstrasse 16, Zürich, Switzerland; 3 Departamento de Física, Faculdade de Ciências, Universidade de Lisboa, Lisboa, Portugal; 4 Centro de Física Teórica e Computacional, Universidade de Lisboa, Lisboa, Portugal; University of Thessaly, GREECE

## Abstract

Worldwide tourism revenues have tripled in the last decade. Yet, there is a gap in our understanding of how *distances* shape peoples’ travel choices. To understand global tourism patterns we map the flow of tourists around the world onto a complex network and study the impact of two types of distances, geographical and through the World Airline Network, a major infrastructure for tourism. We find that although the World Airline Network serves as infrastructural support for the International Tourism Network, the flow of tourism does not correlate strongly with the extent of flight connections available worldwide. Instead, unidirectional flows appear locally forming communities that shed light on global travelling behaviour since there is only a 15% probability of finding bidirectional tourism between a pair of countries. We find that most tourists travel to neighbouring countries and mainly cover larger distances when there is a direct flight, irrespective of the time it takes. This may be a consequence of one-way cyclic tourism that we uncover by analysing the triangles that are formed by the network of flows in the International Tourism Network.

## Introduction

Human evolution has always been marked by mobility, starting with the first *Homo erectus* who began to disperse from Africa soon after their emergence in a journey for survival [1, 2]. Historically, movement of peoples has laid the foundations for exploration and growth: settlements came up, villages became towns, and towns turned into cities [3]. Nowadays, human travel is primarily about business and tourism and relies on transportation networks which are either local, regional or global.

In the last decade, tourism revenues have increased from US$495 billion in 2000 to US$1340 billion in 2017, while international tourist arrivals have grown from 674 to 1322 million in this period and are expected to reach 1.8 billion by the year 2030, according to industry projections [4]. While geographically expanding tourism trends indicate the growth of cultural exploration with time [5], there is a gap in our understanding of how demand for tourism and supply through infrastructure systems like the World Airline Network (WAN) codevelop. As distance decay theory suggests, demand in tourism peaks at neighbouring destinations and exponentially decays farther away [6]. Thus, it should allow a baseline to make predictions on growing tourism worldwide and provide clear strategies for shaping global tourism markets based on geolocated tourism flows. However, the effect of distance on people’s choices is often moderated by their motivations to travel, which have evolved [7]. Nicolau *et al*. explain with deliberation the positive and negative influences of various motivations in deciding travel routes based on distance. Additionally, transport infrastructure plays a significant role in developing destinations and driving tourism internationally [8, 9]. The wan is arguably the largest global transportation network, serving business and tourism needs of the world and enables travelling longer distances with direct flights around the world. This discussion prompts us to question what is the impact of *perceived* distances, which could be affected by the underlying transport system through long-range direct connections, in the decision-making process of tourists.

The representation of the International Tourism Network (ITN) as a complex system allows us to use methods and insights from complex network analysis and data science to investigate the flow of international tourism in a quantitative way [10, 11]. Simultaneously, this formulation also aids in analyzing the structural differences of the tourism network [12, 13] with an omnipresent infrastructure network like the WAN that can, in principle, enable one to travel anywhere in the world. A large number of real-world systems, from social to biological, infrastructural, and financial ones [14–21], have also been studied successfully using network science to classify their structure and dynamic behaviour [22–26].

The goal of this article is twofold. First, we introduce the concept of perceived distances in the form of a quantity called *chemical* distance, which contextualises the tourism network of the world in light of the transportation infrastructure available. We study how patterns emerge in the global tourism market based on geographical distances and compare that to perceived distances. We then characterise the role of distances in the decision-making process of tourism behaviour. Second, a cumulative trend in the choice of destinations worldwide helps us discern how perceived distances can drive demand and supply that dictate origin-destination pairs in tourism. On that basis, we provide possible implications of this study for shaping the development of tourism markets through the WAN.

## Materials and methods

### Datasets

The ITN data was provided by the World Tourism Organization (UNWTO) and consists of the origin and number of “arrivals of non-resident tourists at the national border” from 2004 to 2008 (inclusive) for each country. These overnight visitors are people who travel to a country other than their country of residence, but outside their usual environment, for a period not exceeding 12 months and whose main travelling purpose does not concern any activity remunerated by the country visited [4]. The sources and collection methods differ across countries, varying from border statistics (police and immigration) and supplemented by border surveys, to lodging establishments (i.e., hotels and resorts etc). catered to tourism and standardized by UNWTO. The dataset only considers overnight visitors, i.e., if a person from country *A* is travelling to *B* and takes a connecting flight in *C* but does not stay there overnight, they will only count as a tourist in country *B*, and not in *C*. For smoothing statistical fluctuations, we analyze an agglomerated set over all the years available from the data. This data also cover several years, allowing us to study the time-dependent properties. The construction of the network warrants that self-loops are not present as we are aggregating all airports of one country into one node, thereby removing the necessity of analysing the travel behaviour within a country. This suggests to focus our research question on measuring the impact of inter-country distances on tourism without bias.

The data for the WAN used in this analysis is extracted from *OpenFlights* [27]. It consists of roughly 3500 airports across the 214 countries of the ITN and over 6000 flight connections. The dataset itself has cargo flights as well but for our analysis, we only look at flights that have greater than one commercial passenger (business, tourism or miscellaneous). We start with a coarse-grained version of the WAN at the country level, i.e. each airport of a country is aggregated to one node and links are aggregated between countries (all calculations with the WAN refer to its original state and simplification to the country level has only been done to establish the WTN). Then, we consider the countries present in the WAN as nodes of the ITN and add a link between them if there is a flow of tourists from one country to another. In the simplified WAN if a flight exists from country *A* to *B*, so there must exist a corresponding one in the reverse direction and through the same path. In the ITN, this is not the case as tourism is not necessarily a reciprocal phenomenon. We classify the typology of the network links by the reciprocity of tourism flow, i.e., if there is a two-way flow between the nodes, the link joining them is undirected, otherwise, if the direction of the flow is relevant, the links are marked directed. Furthermore, we add weight to the links using the intensity of tourism flow along the path.

### Distances

We define a quantity of perception for distance, called the chemical distance, as the minimum number of flights required between two countries using the WAN).

In order to calculate the geographical distance between two countries we used the *Haversine distance formula* [28] given by:

$$\begin{array}{c} d=2R\;sin^{-1}(\sqrt{\sin^{2}(\frac{\phi_{2}-\phi_{1}}{2})+cos\left(\phi_{1}\right)\;cos\left(\phi_{2}\right)\;sin^{2}(\frac{\lambda_{2}-\lambda_{1}}{2})}), \end{array}$$ (1)

where *d* corresponds to the geographical distance, *R* to the Earth’s radius, *ϕ*_1_, *ϕ*_2_ to the latitude of points 1 and 2 and λ_1_, λ_2_ to their longitude, respectively. Eq 1 gives the orthodromic distance between two points on Earth. We used the centroid (geographical centre) of each country to make the calculations of the geographical distance between countries. Note that in S1 Fig, we show that the distance distribution of airports to the centroid of their country is ≈ [200, 600] km, significantly smaller than the flight distances in the wan. Most prominent airports fall under this range, exceptions being noted for Russia at 2600 km and the United States at 1600 km. More importantly, for small countries, the airport-to-centroid distances are so small, close to 500 km range on average, compared to the outliers, that our results do not differ when locations of predominant airports are used.

### Weighted reciprocity

To quantify the average ratio of tourism flow ($f_{ij}^{t}$) between nodes, we define the weighted reciprocity as,

$$r^{f}=\frac{1}{L_{\begin{array}{l} 2-way\\ pairs \end{array}}}\underset{i}{\sum }\sum_{\begin{array}{c} j>i\\ j\in \left\{n_{i}^{out}\right\} \end{array}}\{\begin{array}{cc} \frac{f_{ij}^{t}}{f_{ji}^{t}} & f_{ij}^{t}<f_{ji}^{t}\\ \frac{f_{ji}^{t}}{f_{ij}^{t}} & f_{ji}^{t}<f_{ij}^{t}, \end{array}$$ (2)

where $\left\{n_{i}^{out}\right\}$ is the set of countries receiving tourists from *i* and $L_{2-waypairs}$ is the number of bidirectional links with tourism flow in both directions.

### Cyclic clustering coefficient

The *cyclic clustering coefficient* is defined as,

$$\begin{array}{c} C_{i}^{cyc}=\frac{N_{i}^{cyc}}{n_{i}^{D}\left(n_{i}^{D}-1\right)}\;, \end{array}$$ (3)

where $N_{i}^{cyc}$ represents the number of cycle-triangles involving node *i* and $n_{i}^{D}$ represents the number of potential cyclic links. The weighted clustering coefficient is $C_{w}=\frac{\text{total}\;\text{value}\;\text{of}\;\text{closed}\;\text{triangles}}{\text{total}\;\text{value}\;\text{of}\;\text{triangles}}$, in which a triangle is a trio of nodes which is closed if all nodes are connected [29].

### Tourism flow distributions

The distribution of the country-wide outgoing fraction of tourism flow for all chemical distances ($d_{ij}^{c}$) is expressed as,

$$\begin{array}{c} f_{ij}^{t}\left(d_{ij}^{c}\in \left[1,4\right]\right)=\frac{F_{ij}^{t}}{\sum_{j\in \{n_{i}^{out}\}}F_{ij}^{t}}, \end{array}$$ (4)

where $F_{ij}^{t}$ is the number of tourists from *i* to *j*.

Similar to Eq 4, the distribution of the average incoming flow of tourists for all chemical distances ($d_{ij}^{c}$) is expressed as,

$$\begin{array}{c} f_{j}^{t}\left(d_{ij}^{c}\in \left[1,4\right]\right)=\frac{\sum_{i\in \{n_{j}^{in}\}}F_{ij}^{t}}{n_{j}^{in}}, \end{array}$$ (5)

where $n_{j}^{in}$ is the number of in-neighbours of node *j*.

### Network balance

A measure of the structural and functional balance of tourists, *B*^*s*^ and *B*^*t*^, takes into account the degree and tourist traffic from (to) each node,

$$\begin{array}{c} B_{i}^{s}=\frac{k_{i}^{in}-k_{i}^{out}}{k_{i}^{in}+k_{i}^{out}}, \end{array}$$ (6)

$$\begin{array}{c} B_{i}^{t}=\frac{F_{i}^{in}-F_{i}^{out}}{F_{i}^{in}+F_{i}^{out}}, \end{array}$$ (7)

where $F_{i}^{in(out)}$ is the number of incoming (outgoing) tourists from (to) node *i*.

## Results

In order to understand the role of distances in the emergence of tourism, we first view tourism in the context of a network that is formed by tourist flows between countries. The ITN comprises of *N* = 214 nodes (countries) and *L* = 4148 directed links (flow of tourists), with a sparse link density of

$$\begin{array}{c} \frac{L}{N(N-1)}=0.091, \end{array}$$ (8)

with only 9.1% of directed pairs of countries in the network enabling tourism. Note in S2 Fig that the ITN is heterogeneous like an Erdös-Réyni graph [30], while the WAN follows a truncated power-law distribution with a decay function [31]. The average number of countries visited by residents of a country is given by the average out-degree,

$$\begin{array}{c} <k^{out}>=\frac{\sum_{i}^{N}k_{i}^{out}}{N}=19.38, \end{array}$$ (9)

with a standard deviation value of *σ*_<*k*^*out*^>_ = 11.67. Since all links in the ITN have a start and end node, the total in- and out-degree are the same, as are their averages. The average out-degree value indicates that the outgoing tourism flow is not widespread and its standard deviation reflects the disparity in varied tourism patterns of countries. While the average degrees are the same, a much larger deviation in the in-degree of *σ*_<*k*^*in*^>_ = 45.89 explains how tourists originate from many countries around the world but travel to only a few of them. Interestingly, the distribution of the *out-degree* $k_{i}^{out}$ for the ITN is remarkably different from the physical infrastructure of the WAN that enables long-distance tourism in the first place [31].

**Fig 1:**
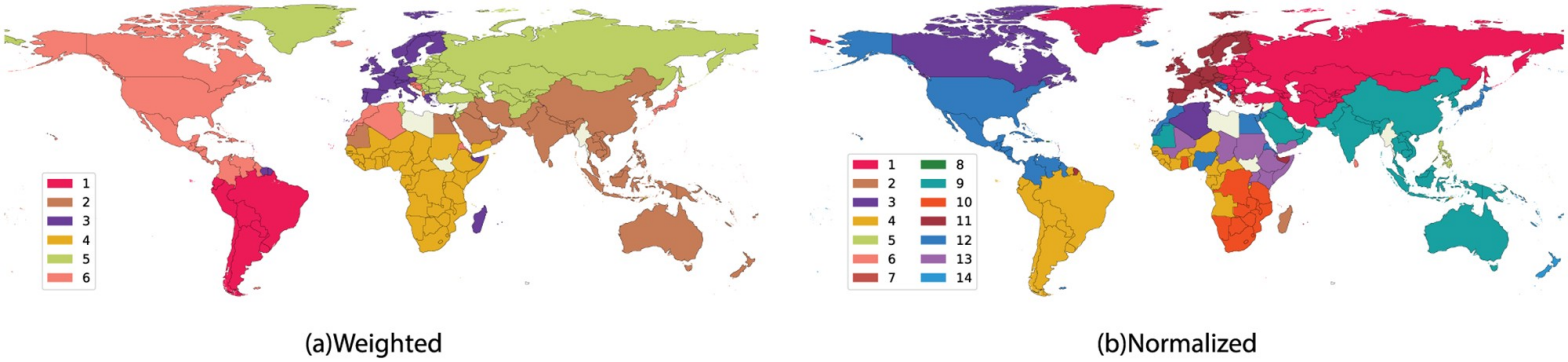
(a) **Weighted Community Structure of the ITN**: Links are weighted by the flow of tourists. The number of communities is six and the modularity value is *Q* = 0.54. (b) **Normalized Weighted Community Structure of the ITN**: Links are weighted as the flow of tourists divided by the population of the sending country. The number of communities is 13 and the modularity is *Q* = 0.7. The different numbers and colours in the figure refer to the communities found by using random sampling of starting conditions for the detection algorithm. The countries for which we have no tourism data are represented in grey. This figure was created by using the open-source software *Gephi*, which is distributed under CDDL 1.0 and GNU General Public License v3 [35].

The different incoming and outgoing link distributions are a strong indication of clusters within international tourism. These clustered regions are typically formed by a limited number of countries between which the flow of tourists is substantially larger than to other territories (see Fig 1(a)). We observe six known communities [32] in different regions of our planet using tourism flow as weights and the standard community detection method [33]: Western Europe, Eastern Europe, Middle and Far-East with Oceania, South America, North America and Africa. Certain exceptional connections involving Suriname, French Guyana and Madagascar within the central European community (Fig 1(a)) may point to stronger tourism ties stemming from a colonial history [34]. Considering every country has a different scale of outgoing tourism, we analyze the communities by using rescaled weights of tourism flow by the population of the originating country. Fig 1(b) illustrates that people prefer to travel to countries within their continent or sub-continent, even though the effects of globalization have created an intricate network of airlines around the world with intercontinental links making up 68.08% of the ITN. There are a few exceptions though: community 12 shows that flows among the United States of America, Nigeria, Morocco and Egypt, normalized by population, are stronger than outside the community. We reckon this is a population bias that clusters the countries together. On the other hand, community *4* is uncovering a tourism trend between South America and Western Africa, which may be because of its wildlife economy (see S1 Table and S3 Fig for the evolution of tourism ties). Communities are robust and were extracted by random sampling of starting conditions for the detection algorithm.

**Fig 2:**
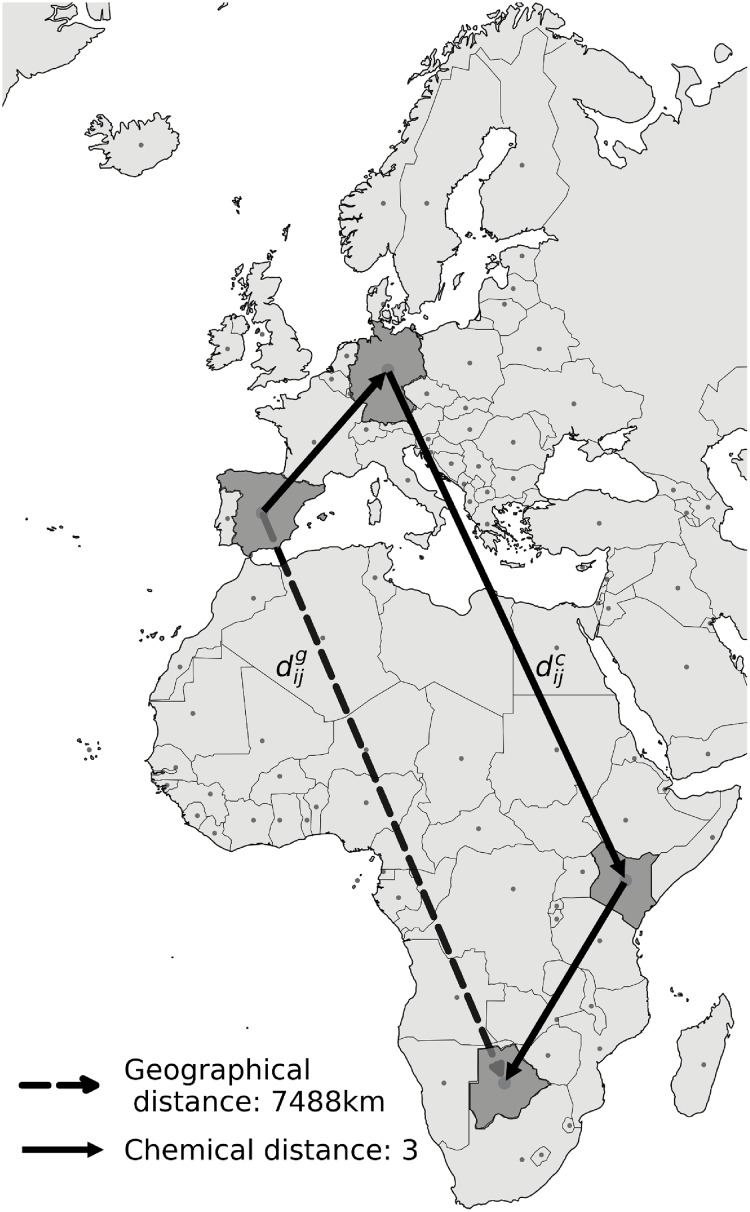
Representation of the ITN. Illustration of the tourism flow from Spain to Botswana, for which the geographical distance $d_{ij}^{g}$ is 7488 km and the chemical distance in the coarse-grained WAN $d_{ij}^{c}$ is 3, illustrating that tourists going from Spain to Botswana have to take at least two inter-country stopovers, travelling, for example, through Germany and Kenya, which is a much larger geographical distance than what separates the two countries. Note that the distance is measured between the centroids of each country. This figure was created by using the open-source software *Gephi*, which is distributed under CDDL 1.0 and GNU General Public License v3 [35].

In the WTN, as a proxy for perceived distances, we define a quantity called the chemical distance $d_{ij}^{c}$ (see Fig 2), which is the least number of flights required to travel from country *i* to *j* over the ITN. We observe that the average shortest path length required by a resident of one country to travel to another is <*l* > = 2.32 with a standard deviation of *σ*_<*l*>_ = 0.69, i.e., on average, a traveller is taking 1.32 connections (stopovers) in order to go from their country to any other. It is a smaller value than what has been previously reported for the WAN [31], and in comparison depicts how tourism is not strongly correlated with the availability of the physical infrastructure in the WAN, which may also be used for business and other needs like family and diplomacy.

For more detailed analyses of the differences in geographical and chemical distances, we study the statistical properties of the two networks in detail. The WAN is topologically bidirectional by construction, i.e. there is almost always a return flight available between a pair of airports [36]. By contrast, since the tourism network has an uneven directed flow of tourists from one country to another, only 15% of the links are bidirectional, i.e., a flow of tourists in both directions. We developed a simple measure of weighted reciprocity as a proxy for the extent of tourism exchange between two countries (see Materials and methods Eq 2) on bidirectional links. *r*^*f*^ = 0.44, which shows that the fraction of tourism flow (on average) on the lesser-used direction is 44% of the flow the other way. A possible explanation for an uneven tourism flow between countries could be due to the heterogeneous nature of the economies of the countries in the ITN and highlights the enormity of an uneven transfer of wealth through tourism. Given the modular structure of tourist flows around the world, repatriation of revenues through tourism and payments for import goods to other countries could add to the leaking of funds earned through tourism [37].

**Fig 3:**
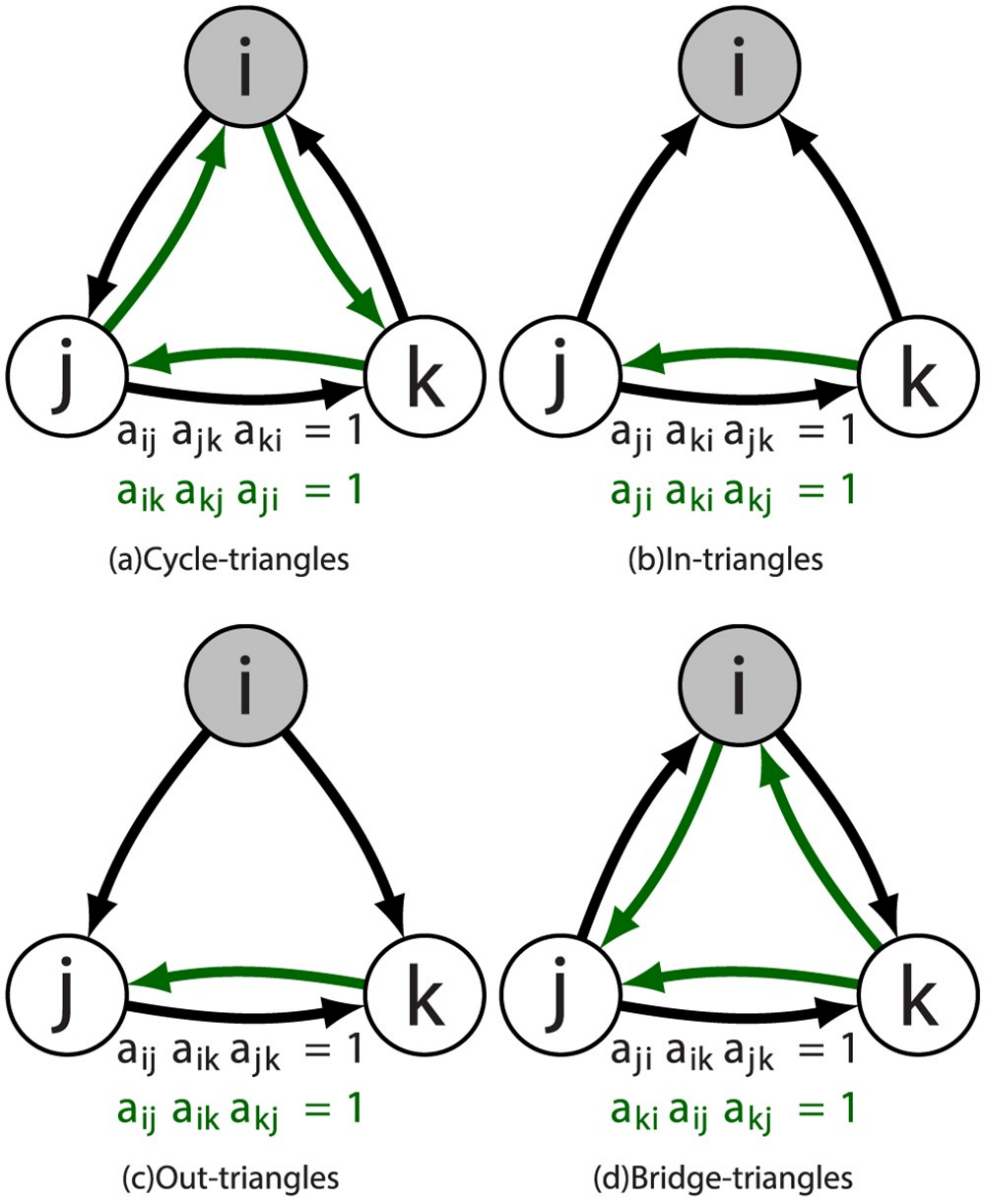
Types of triangles. Triangles in directed networks and their designations. Each type of triangle corresponds to a different product of the adjacency matrix, which is shown below each sub-figure. If there is a flow of tourists from node *i* to *j* then *a*_*ij*_ = 1, else 0.

To carefully understand the concept of leakage in modular communities, we define the flow of tourism wealth using the amount of cyclic clustering that exists in the network for each country in the form of cycles. For undirected networks, there is only one possible triangle formed between each trio of nodes. ITN is, however, directed in its movement of travellers [38], in which case that number increases to eight, as shown in Fig 3. We only consider the cycle-triangles for the calculation of the clustering coefficient since these triangles indicate there is a unidirectional cyclic flow of tourism wealth among countries. Cycle-triangles form cliques of size three, which are triplets of nodes that are all connected among themselves. In a tourism network, cliques would enable countries to transfer wealth back to themselves, thereby host communities replenishing the leakage of their tourism revenues. This phenomenon works particularly well for developing nations [39].

Taking into account these factors, we expect the probability of occurrence of cycles in a directed network to be of the order of the link density, since if a node is connected to two other nodes, the latter only need to form a link between themselves (in the right direction), in order to form a cycle. The average value of cyclic clustering coefficient for the nodes of the ITN is <*C*^*cyc*^ > = 0.015, which is of the order of the link density, as expected. It is, though, of a small order for a network built on top of the WAN, which itself has a high value of clustering (without cycles) (*C* = 0.62 [32]).

The weighted clustering coefficient of the ITN is *C*_*w*_ = 0.0077. If we neglect the direction of the links in the ITN and calculate merely the unweighted and undirected clustering coefficient [40], we obtain <*C*^*und*^ > = 0.79, a much larger value than for the directed version of the network and slightly larger than the WAN itself. This difference asserts our profile of global tourism, in that the world is very well connected in the context of air travel with built-in redundancies in connectivity but flow of tourism is mostly unidirectional, perhaps, exposing the lack of opportunities for equitable tourism globally, where people tend to go from richer countries to poorer ones, contributing to the development of the third world [41].

**Fig 4:**
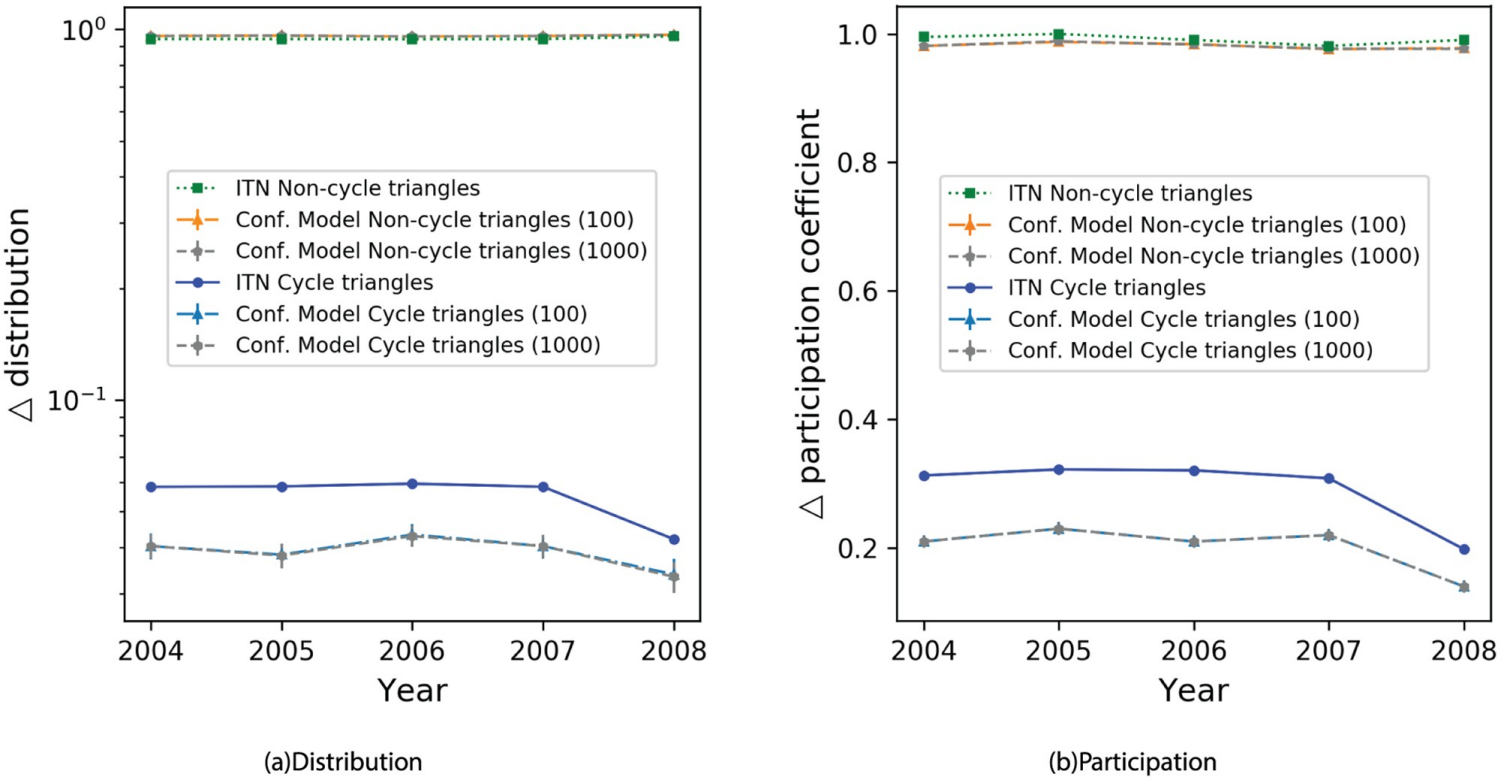
Change in distribution and participation of cycles over time. (a) Triangle distribution values for the ITN and the configuration model networks. (b) Triangle participation coefficient values for the ITN and the configuration model networks. These networks were generated by randomizing the links present in the ITN while keeping the degree distribution of its nodes intact. The results present here are a robust average of 100 and 1000 instances of these networks.

The percentage of cyclic triangles in the ITN is about 6%. The diminished number of cycles add more weight to leakage of revenues through tourism. Moreover, only 25% of the countries inadvertently form at least one cyclic triangle. In order to have a benchmark, we reproduce the *configuration model* by rewiring all the links in a network to generate a new network, which is uncorrelated in terms of node-node correlations, while keeping the degree sequence of the nodes intact [42]. The structural representations of the two networks (see Fig 4) indicate that the distribution of triangles in the ITN resembles the distribution of triangles within a random network as opposed to a real-world network. A detailed tabular report is presented in the S2 Table.

**Fig 5:**
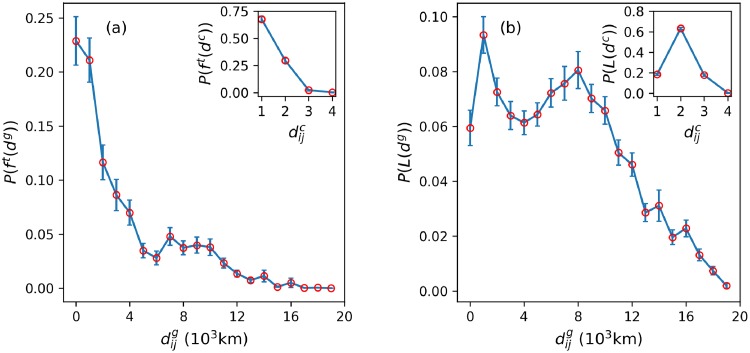
Distribution of tourism and links. (a) Outgoing tourism flow distribution of the ITN using geographical and chemical (inset) distances as spatial indices. (b) Neighbourhood link distribution of the ITN using geographical and chemical (inset) distances as spatial indices. The countries in the neighbourhood of an originating country are the ones that receive tourists from it. The error bars represent the standard error.

While the ITN is formed on the physical linkages of the WAN, its spatial organization differs for both chemical and geographical distances (see Materials and methods). In order to study the influence of the *chemical distance* on global trends in the choice of destinations worldwide, we analyse the distributions of the fraction of tourists as a function of geographical and chemical distances (from 1, a direct flight, to 4, the largest chemical distance in the ITN). We measure the distribution of tourists, $P(f_{ij}^{t})$ (see Materials and methods), as a function of the geographical distance by dividing the distance between countries into 1000 km bins. We notice that the peak of the fraction of tourists is in the (0, 1000] km interval. It is important to note that the first two bins have very similar values, which means that people are almost as likely to travel to a destination that is at most 1000 km away as they are to a destination between 1000 and 2000 km away, and the distribution decays with the distance after. The resulting distribution of the fraction of tourists originating from a country on each chemical link is presented in the inset of Fig 5(a), where we observe that on average, for the entire network, the fraction of tourists is highest on single link segments to neighbouring countries and decreases monotonically with an increase in the number of links between the nodes, thereby indicating that tourists prefer direct links. Approximately 77% of global tourists travel as far as 4000 km. Similarly, Fig 5(b) shows the fraction of neighbours that receive tourists between countries based on geographical distances (again binned by 1000 km) and chemical distances from the originating country.

The distribution of links for chemical distances in the inset of Fig 5(b) suggests that even though most outgoing tourism links from a country on average are at a distance $d_{ij}^{c}=2$, typically more travellers fly to countries that are directly connected for tourism. Geographically speaking, the link distribution does not exhibit a large difference in the values, at least for the (0, 11000] km range, as previously observed for the tourism-share, indicating that there is still some variety in the choice of travelling towards farther countries. More importantly, note that while the probability of finding links does not significantly change concerning geographical distance (0, 8000] km, the network is utilizing the underlying WAN only locally exhibited by the peak in the chemical distance distribution. The distribution is bimodal and the first peak may represent neighbouring countries in terms of geographical distance only, while the second peak points to long-haul flights that may also constitute a stopover (see inset of Fig 5(b) for $d_{ij}^{c}=2$). As a whole, Fig 5 illustrates the average distributions of tourism and links between countries for various distances. Following that, the most amount of traffic moves on a smaller subset of links with $d_{ij}^{c}=1$ and a lesser amount of traffic has a rather large subset of links available due to a variety of travelling behaviour (see inset of Fig 5(b)).

**Fig 6:**
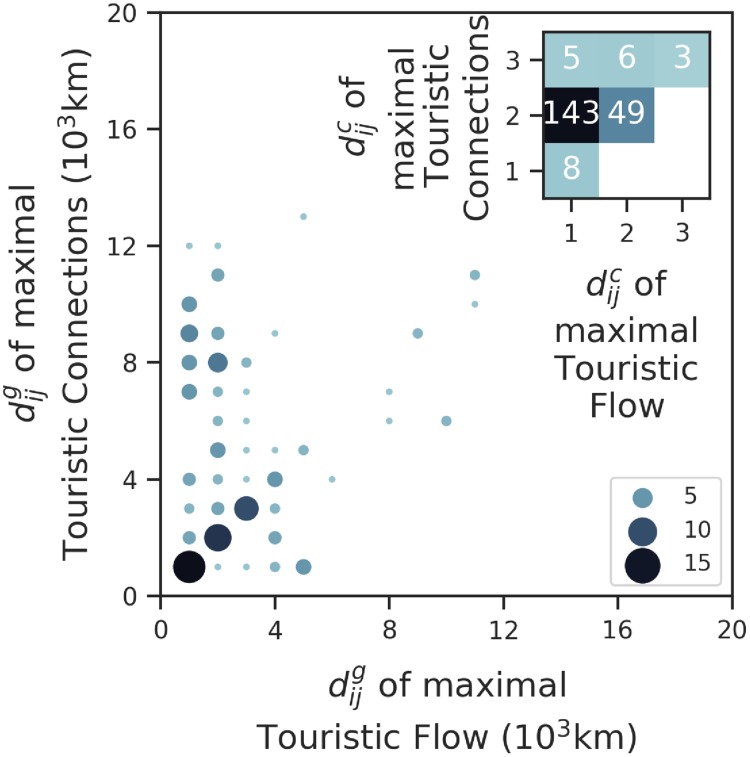
Variability in supply and demand. A binned scatter plot indicates the relationship between geographical distances of each country in the ITN for which the tourism share and number of links is maximal. The squares without a number correspond to unregistered occurrences. In the inset, a heatmap indicates the relationship between chemical distances for which the tourism share and number of links maximizes.

**Fig 7:**
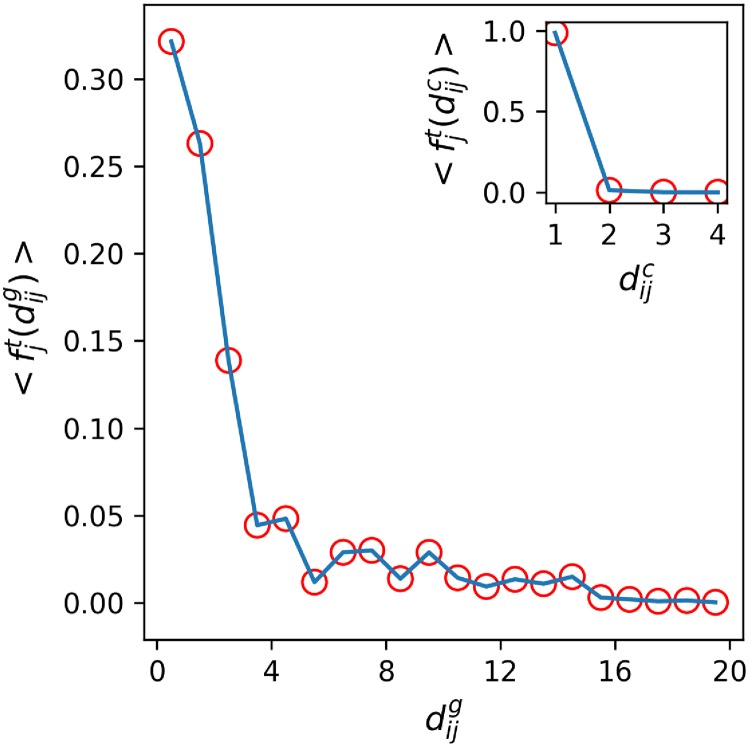
Distribution of tourism and links. Country-wide incoming distribution of average tourism flow of the ITN using geographical and chemical (inset) distances as spatial indices.

Fig 6 compares the distances at which the distributions of tourism share and links maximize for each country. We notice that the demand (tourism flow) and supply (links) variability for geographically binned (1000 km) distances do not correlate. Since the average fraction of tourists from any country that prefer a range of (0, 2000] km on a direct link is 0.44 ± 0.02, there are not enough data points beyond that distance range to report anything statistically significant. However, similar to Eq 4, the distribution of the average incoming flow of tourists, $P(f_{j}^{t})$ for all chemical distances ($d_{ij}^{c}$), is a proxy for the demand being met. Our analysis shows that, on average, a country receives the most number of incoming tourists from a direct link (see Fig 7, normalized over all the points in the plot). The geographical distribution of incoming tourism also shows that countries on average receive roughly 72% of global tourists from (0, 3000] km range.

**Fig 8:**
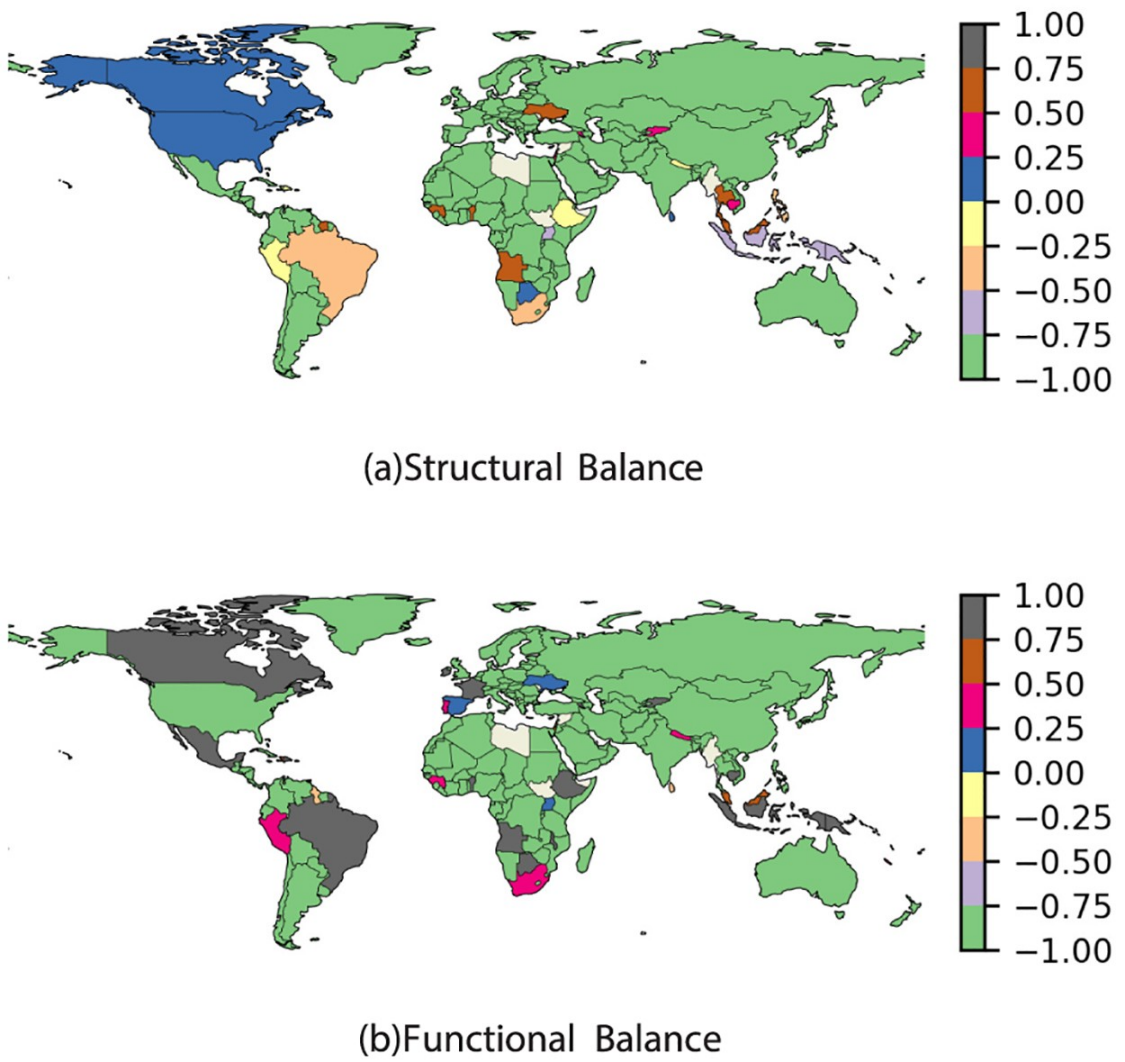
Structural and functional balance. Colour coded map of the countries in the ITN for (a) structural balance $B_{i}^{s}$ and (b) functional balance $B_{i}^{t}$. The majority of countries are in the [−1, −0.75] region indicating that most tourism is outgoing in nature. The countries for which we have no tourism data are represented in grey. This figure was created by using the open-source software *Gephi*, which is distributed under CDDL 1.0 and GNU General Public License v3 [35].

**Fig 9:**
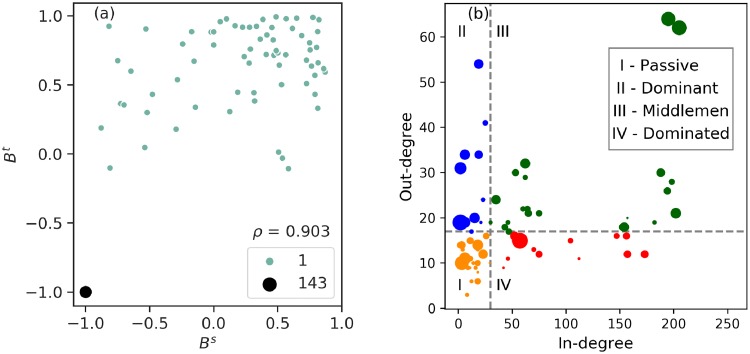
Categorical balance. (a) Scatter plot of the *B*^*t*^ and *B*^*s*^ parameters. Their correlation coefficient is *ρ* = 0.903 which means that countries typically have similar structural and functional balance. (b) 50%-Quantile division of the in and out-degree of the countries present in the ITN with a colour code respective to their strength of bi-directional tourism (I—Passive, II—Dominant, III—Middlemen, IV—Dominated). The radius of each marker is proportional to the ratio of its outgoing and incoming tourist flow. Note: we did not include the nodes with zero in degree in the figure, for the visualization to not have an infinite ratio for size.

Certainly, the network is not in balance, i.e. the reciprocity of tourism is not bidirectional and equal. The disparity in the flow distribution, however, is not a consequence of the connectivity of regions because these links form the physical infrastructure of the WAN which is universally reciprocal. So, which nodes in the network are tipping the scales of this balance? We measure the structural and functional balance of tourists, *B*^*s*^ and *B*^*t*^, by taking into account the degree and tourist traffic from (to) each node (see Materials and methods). By definition, both $B_{i}^{s}$ and $B_{i}^{t}\in \left[-1,1\right]$. Most countries have a value −1, in that there is a strict flow of outgoing tourists only, while 1 indicates countries with a strictly incoming flow. The distribution of the structural and functional balance throughout the network is illustrated in Fig 8(a) and 8(b). Note that most countries are in the region of [−1, −0.75] indicating that tourism is, in general, more outgoing than incoming. Furthermore, the two quantities of balance are correlated with *ρ* = 0.903 as shown in Fig 9(a). Since the hypothesis underlying the correlation plot has 143 points with a zero or unreported balance measure, there is no significance in noting the *p-value*. Fig 9(b) shows that the four quadrants in the 50%-quantile division plot are not equally spaced out; most nodes have a low incoming degree, thereby reinforcing our analysis. Thus, by analysing the distributions of tourism traffic, the variability of traffic on chemical links and the balance in the network, we can discover the unequal flows of tourism worldwide.

## Conclusion

In this article, we study the effect of distances on tourism and how perception changes from small to large distances. Our results show that even though outbound tourism is mostly directed towards closer destinations than farther ones, adding long flights to the underlying infrastructure network poses a significant advantage in shaping perceived distances of travellers. The relationship between the WAN and the ITN sheds light on how the two structures are very dissimilar and have likely codeveloped. In fact, tourism demand seen in the ITN is not always matched by the supply network of the WAN indicating that business demands also form a large part of the infrastructure supply, i.e., the decaying distribution of flow of tourists is most likely a result of decaying motivation to travel longer distances for tourism without the availability of direct flights.

The airline network is significantly different from the network of travellers around the world. Most countries do not experience tourism in large numbers or from diverse places. Even though the WAN offers flight connections to get to everywhere in the world, a statistically quantitative understanding of emerging community patterns illustrates that tourism is largely intracontinental with a few exceptions where new connections may be implemented due to changing cultural patterns in exploration (such as long-distance flights). While McKercher *et al*. establish compelling evidence for communities forming based on geographical distance [6], these exceptions set the stage for probing how distances are perceived in the first place in shaping global tourism.

Recently, increasing tourism revenues have prompted airlines to add long-distance and cheap flights to their fleet, focussing on fast-paced tourism. Typically, these flights aim at direct connections and in some cases, allow for a few hops to reach farther destinations in-country. This is a classic case of co-development as adding a flight to a previously disconnected destination also attracts more customers, possibly by reducing their perceived distance of the destination. Once in the destination country, alternate modes of transport also provide for a larger tourism sprawl.

While new ties transfer wealth from one country to another, the international tourist network is not balanced by reciprocal flow in both directions. Normally, tourists from richer countries visit poorer ones. Besides, repatriation of profits often results in weakening of economies of countries being visited. Our findings related to cyclic tourism structures in the ITN leads us to believe it is imperative to further explore how wealth is transferred through tourism as there is strong evidence that communities exist within the ITN. As the role of distance and perceived distance becomes more clear for tourism, it may be possible to design optimization strategies for the WAN to develop countries that need a boost in their economy and are willing to provide a sustainable experience to travellers. It is also important to meet the demands of the high season of tourism by using a variable underlying airline network, in which, flight patterns could be adjusted depending on seasonal requirements for reducing the negative impacts of flying with empty long-distance aircraft.

## Supporting information

S1 Table Change of tourism flow between 2004 and 2008. (PDF)

S2 Table Triangle decomposition of the ITN and configuration model networks. (PDF)

S1 Fig Airport to centroid distance. A boxplot showing the distribution of airport distances to the centroid of their respective country, for the world and 4 representative countries in the WAN dataset.
(TIF)

S2 FigComparison of the out-degree of ITN and the WAN.The average out-degree for the ITN is $k_{i}^{out}=19.38$ and its standard deviation has a value of *σ*_<*k*^*out*^>_ = 11.67. The distribution of the *out-degree* parameter shows a deviation from a power-law distribution, which would be expected for a real network like the WAN where the distribution is of power-law with an exponential cut-off [31]. (a) Degree distribution of the ITN and an Erdős–Rényi model graph with the same number of nodes and with edge probability *p* = 0.91, the same as for the ITN. (b) Cumulative degree distribution of the WAN and a Barabási–Albert network with each node connected to *m* = 5 other nodes.(EPS)

S3 Fig Change of tourism. Distribution of links in the ITN in terms of their *α* parameter value with *t*_0_ = 2004 and *t*_*n*_ = 2008. In the inset, we plot the <*α*_*ij*_> over the years of the dataset.
(EPS)
