# Supplementary material for: Impact of perceived distances on international tourism
Source: PLoS One. 2019 Dec 4;14(12):e0225315. doi: 10.1371/journal.pone.0225315 (PMC6892543; doi:10.1371/journal.pone.0225315)
Supplement: S1 Table — (PDF) [file pone.0225315.s001.pdf]

S1 Table

Alpha parameter for links that have evolved the most and least between $2004 - 08$ A simple measure of the strength of evolution of tourism flow α_{ij} between a pair of countries i and j is defined as,

$$\alpha_{ij}(t_n) = \frac{F^t_{ij}(t_n) - F^t_{ij}(t_0)}{F^t_{ij}(t_0)}, \tag{1}$$

where t_n and t_0 correspond to the end and start of discrete-events in the network dataset (2008 and 2004, respectively), shows that big changes in flow have occurred for a very few links over the years (trailing tail) and $\approx 30\%$ of the links have shown no change in traffic.

Table 1. Combination of countries representing a change of tourism flow between them. The top half illustrates the links that have strengthened from 2004 to 2008. The bottom half are links that have weakened over time. The value measure is α, a parameter to showcase the change in the flow of tourism from 2004 to 2008 in the International Tourism Network (ITN).

Origin country	Destination country	α_{ij}
Macedonia	Israel	881
Laos	Cambodia	35.75
Tajikistan	Kyrgyzstan	35.23
Rwanda	Ukraine	26.11
Uzbekistan	Kyrgyzstan	17.66
Kuwait	Israel	-0.93
Georgia	Trinidad and Tobago	-0.96
United Arab Emirates	Israel	-0.96
Saudi Arabia	Israel	-0.99
Macau	Malaysia	-0.99
