# Supplementary material for: Impact of perceived distances on international tourism
Source: PLoS One. 2019 Dec 4;14(12):e0225315. doi: 10.1371/journal.pone.0225315 (PMC6892543; doi:10.1371/journal.pone.0225315)
Supplement: S2 Table — (PDF) [file pone.0225315.s002.pdf]

S2 Table

Triangle decomposition of the International Tourism Network (ITN) and configuration model networks. The triangle distribution indicates the frequency of each type of triangle in the network, while the triangle participation tells us how many nodes belong in each type of triangle. The configuration model networks were built by randomizing the links present in the ITN while keeping the degree distribution of its nodes intact. The results were obtained after simulating 100 instances of these networks and taking the average of each parameter.

Table 1. Triangle distribution and participation values for the ITN and configuration model networks. These networks were created by randomizing the links present in the ITN while keeping the degree distribution of its nodes intact. The results present here are obtained after simulating 100 instances of these networks and taking the average of each parameter.

		Cycle	In	Out	Bridge
Triangle Distribution	ITN*	5.89%	31.37%	31.37%	31.37%
	Conf. Model	4.66(±0.29)%	31.78(0.1)%	31.78(0.1)%	31.78(0.1)%
Triangle Participation	ITN*	32.24%	31.77%	100%	32.24%
	Conf. Model	22.3(0.5)%	22.3(0.5%)	99.7(0.3)%	22.9(0)%
