# Supplementary figures and images for: Impact of perceived distances on international tourism
Source: PLoS One. 2019 Dec 4;14(12):e0225315. doi: 10.1371/journal.pone.0225315 (PMC6892543; doi:10.1371/journal.pone.0225315)

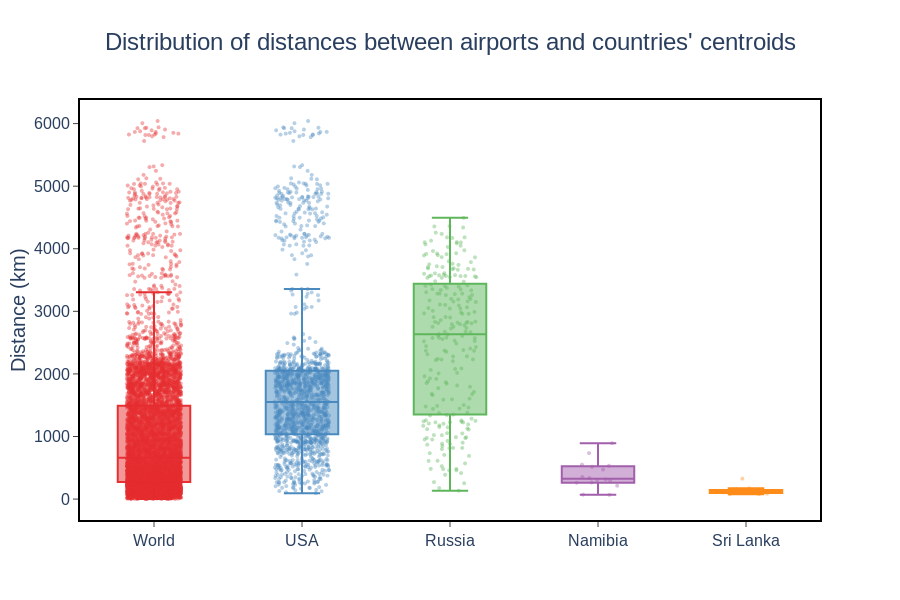

Supplement: S1 Fig — A boxplot showing the distribution of airport distances to the centroid of their respective country, for the world and 4 representative countries in the WAN dataset. (TIF) [file pone.0225315.s003.tif]
